# Cells Derived from the Coelomic Epithelium Contribute to Multiple Gastrointestinal Tissues in Mouse Embryos

**DOI:** 10.1371/journal.pone.0055890

**Published:** 2013-02-13

**Authors:** Rita Carmona, Elena Cano, Andrea Mattiotti, Joaquín Gaztambide, Ramón Muñoz-Chápuli

**Affiliations:** 1 Department of Animal Biology, Faculty of Science, University of Málaga, Málaga, Spain; 2 Pediatric Surgery Unit. Maternity and Children Hospital, Regional Hospital Carlos Haya, Málaga, Spain; Northwestern University, United States of America

## Abstract

Gut mesodermal tissues originate from the splanchnopleural mesenchyme. However, the embryonic gastrointestinal coelomic epithelium gives rise to mesenchymal cells, whose significance and fate are little known. Our aim was to investigate the contribution of coelomic epithelium-derived cells to the intestinal development. We have used the transgenic mouse model mWt1/IRES/GFP-Cre (Wt1^cre^) crossed with the Rosa26R-EYFP reporter mouse. In the gastrointestinal duct *Wt1,* the Wilms’ tumor suppressor gene, is specific and dynamically expressed in the coelomic epithelium. In the embryos obtained from the crossbreeding, the *Wt1*-expressing cell lineage produces the yellow fluorescent protein (YFP) allowing for colocalization with differentiation markers through confocal microscopy and flow cytometry. Wt1^cre-YFP^ cells were very abundant throughout the intestine during midgestation, declining in neonates. Wt1^cre-YFP^ cells were also transiently observed within the mucosa, being apparently released into the intestinal lumen. YFP was detected in cells contributing to intestinal vascularization (endothelium, pericytes and smooth muscle), visceral musculature (circular, longitudinal and submucosal) as well as in Cajal and Cajal-like interstitial cells. Wt1^cre-YFP^ mesenchymal cells expressed FGF9, a critical growth factor for intestinal development, as well as PDGFRα, mainly within developing villi. Thus, a cell population derived from the coelomic epithelium incorporates to the gut mesenchyme and contribute to a variety of intestinal tissues, probably playing also a signaling role. Our results support the origin of interstitial cells of Cajal and visceral circular muscle from a common progenitor expressing anoctamin-1 and SMCα-actin. Coelomic-derived cells contribute to the differentiation of at least a part of the interstitial cells of Cajal.

## Introduction

Intestinal motility disorders include a collection of pathologic processes secondary to alterations in the intestinal wall. These alterations may be congenital or secondary to degenerative changes due to systemic illness. Congenital disorders in the development of the enteric nervous system have been described in Hirschsprung disease, intestinal neuronal dysplasia, ganglioneuromatosis or chronic intestinal pseudoobstruction. Familial visceral myopathy is characterized by alterations in the smooth muscle fibers of the muscular layers of the intestinal wall causing a clinical picture of chronic intestinal pseudoobstruction. Some of these pathologies have been correlated with alterations in the network of interstitial cells of Cajal (ICC) [1]. Despite the potential importance of congenital factors in the pathogeny of these anomalies, the knowledge on the embryonic development of the mesodermal cell populations that contribute to the different intestinal tissues is still insufficient.

The coelomic epithelium of the mammalian embryo is composed of a single, squamous cell layer that invests the external surface of all the viscera and the inner surface of the body wall. The embryonic coelomic epithelium has recently revealed to be a very active tissue participating in visceral organogenesis. This is due to its ability to generate mesenchymal cells through a process of epithelial-mesenchymal transition (EMT). In this way, the embryonic epicardium gives rise to epicardially-derived cells that contribute to the cardiac vascularization and also play a key signaling role for myocardial growth and differentiation [2]–[4]. Migration of coelomic cells into the lung buds to give rise to vascular smooth muscle, has also been reported [5]. The mesothelium of the developing liver is also involved in the origin of sinusoidal endothelial and stellate cells [6]. In the testicles, progenitors of Sertoli cells migrate from the coelomic epithelium of the genital ridges [7].

However, the role played by the coelomic epithelium of the intestine during its development is far less known. The digestive tract develops by association of the endodermal tube with the surrounding splanchnic mesenchyme, which is later colonized by the neural crest cells [8]. It has been reported that, as in other viscera, the coelomic epithelium transforms into migrating cells which incorporate to the mesenchymal compartment and contribute at least to the vascular smooth muscle [9]. These authors used the Wilms’ tumor suppressor gene *Wt1* as a lineage marker of the coelomic and coelomic-derived cells. In fact, *Wt1* is dynamically expressed in the coelomic epithelium as well as in coelomic epithelium-derived cells in many organs [10]. We have developed a model based on the same mWt1/IRES/GFP-Cre (Wt1^cre^) recombinase system from other studies [11], [12] but using the Rosa26R-EYFP mouse line as reporter. In our hands, this model has shown a much higher sensitivity than the Rosa26R-LacZ reporter. Cells that express YFP in the reporter line after recombination with the Wt1^cre^ mouse (herein referred to as Wt1^cre-YFP^ cells) could be easily immunolabelled with a number of differentiation markers. This has allowed us to describe how coelomic epithelium-derived cells play multiple and hitherto little-known roles in intestinal development and contribute to many cell populations. The embryonic origin of two of these populations, Cajal and Cajal-like interstitial cells (ICC and ICC-like, respectively), was poorly known. ICC-like have been described as cells closely related to ICC in the gut, but lacking of c-Kit expression [13]. Their embryonic origin and precise function are unknown. ICC are closely associated to the gut musculature and neurons and they act as pacemakers for gastrointestinal contractility [14]. The hypothesis of a common progenitor for ICC and the visceral musculature has received experimental support [15]. This hypothesis is supported by the findings herein shown, that also point out to the role played by coelomic-derived cells in these developmental processes.

## Methods

The animals used in our research program were handled in compliance with the institutional and European Union guidelines for animal care and welfare. The experimental procedures were approved by the Committee on the Ethics of Animal Experiments of the University of Málaga (procedure code 2009–0037).

The mWt1/IRES/GFP-Cre (Wt1^cre^) transgenic mouse line is the same used for previous studies of the Wt1 lineage [11], [12]. The endogenous expression of GFP in embryos of this line was not detectable by confocal microscopy after the fixation procedure used in our study. Homozygote (Cre+/+) mice were crossed with Rosa26R-EYFP (B6.129X1-Gt(ROSA)26Sortm1(EYFP)Cos/J). Both homozygote mouse lines were maintained and bred at the UMA facility.

Embryos were staged from the time point of vaginal plug observation, which was designated as the stage E0.5. Whole embryos and the viscera of neonates were excised, washed in PBS and fixed in 4% fresh paraformaldehyde solution in PBS for 2–8 h. Then, the embryos were washed in PBS, cryoprotected in sucrose solutions, embedded in OCT and frozen in liquid N_2_-cooled isopentane. Ten µm cryosections were stored at −20°C until use.

Immunofluorescence was performed using routine protocols. Cryosections were rehydrated in Tris-PBS (TPBS) and blocked for non-specific binding with SB (16% sheep serum, 1% bovine albumin in TPBS) or SBT (the same solution plus 0.1% Triton X-100) for membrane-bound and intracellular antigens, respectively. When biotinylated secondary antibodies were used, endogenous biotin was blocked with the Avidin-Biotin blocking kit from Vector. Single immunofluorescence was performed incubating the sections with the primary antibody overnight at 4°C, washing in TPBS and incubating with the corresponding fluorochrome-conjugated secondary antibody. Secondary antibodies were not used in the case of the anti-CD34 antibody, which was conjugated to eFluor660. Nuclei were counterstained with DAPI (Sigma). Double immunofluorescence was performed by mixing both primary antibodies (rabbit polyclonal and mouse or rat monoclonal), and incubating overnight at 4°C. We then used a Cy5-conjugated and a biotin-conjugated secondary antibody, followed by a 45 min incubation with TRITC-conjugated streptavidin. No nuclear counterstaining was made on these slides. In the case of the double CD34/SMCα-actin immunostaining we incubated overnight the sections with the anti-CD34 antibody conjugated to eFluor660, then we blocked the sections with monovalent donkey anti-mouse IgG or Mouse-on-Mouse blocking kit (Vector), and we incubated the sections again with the anti-SMCα-actin antibody. TRITC-conjugated rabbit anti-mouse IgG was used as secondary antibody. C-kit immunostaining was performed on paraformaldehyde-fixed intestinal tissue cryosections using a polyclonal anti-c-kit antibody (Dako) and a secondary antibody (Cy5-conjugated donkey anti-rabbit IgG). Negative controls were always performed incubating with non-immune rat isotype, mouse or rabbit IgG instead of the primary antibody.

Wt1 immunohistochemistry was performed on unfixed, cryoprotected embryos frozen as described above. Cryosections were fixed in cool methanol/acetone (1∶1) for 10 min. The sections were incubated with a rabbit polyclonal anti-Wt1, biotinylated anti-rabbit IgG and extravidin-peroxidase. Finally they were revealed with a diaminobenzidine kit (Sigma-Aldrich).

Details of the antibodies used in this study are provided in Table 1.

**Table 1:** Antibodies used in this study.

Antibody	Supplier	Clone or Ref.	Dilution
Monoclonal rat Anti-mouse CD31 (PECAM)	Pharmingen	Ref. 550274	1/20
Monoclonal mouse anti alpha smooth muscle actin	Sigma	Clone 1A4 Ref. A2547	1/100
Monoclonal rat Anti mouse CD117 (c-kit)-APC conjugated	eBioscience	Ref. 17-1172	1/20
Monoclonal rat Anti-mouse CD34 eFluor 660	eBioscience	Ref. 50-0341	1/100
Rabbit polyclonal anti-pan cytokeratin	Dako	Ref. Z0622	1/100
Rabbit polyclonal anti- human c-Kit	Dako	Ref., A4502	1/100
Rabbit polyclonal anti-laminin	Sigma	Ref. L9393	1/200
Rabbit polyclonal anti-alpha NG2	Abcam	Ref. ab 5320	1/50
Rabbit polyclonal anti-FGF9	Antigenics	Ref. RNF 324	1/50
Rabbit polyclonal anti-RALDH2	Gifted by Peter McCaffery(Univ. of Massachussets)		1/2000
Rabbit polyclonal anti-ANO1	Abcam	Ref. ab53212	1/20
Mouse monoclonal anti-5B5 (prolin-hydroxylase subunit)	Abcam	Ref. ab 44971	1/50
Rabbit polyclonal anti-fibronectin	Sigma	Ref. F3648	1/100
Rabbit polyclonal anti-FSP1	Gifted by Dr. Eric Neilson (VanderbiltUniv. School of Medicine)		1/100
Rabbit polyclonal anti- PDGFR alpha	Abcam	Ref. 61219	1/200
Rabbit polyclonal anti-Wt1	Santa Cruz	Sc-192	1/100

For flow cytometry analysis, intestines from Wt1^cre-YFP^ embryos were excised, dissociated for 15 min at 37°C in pre-warmed Accutase (Millipore) and homogenized by repeated pippeting. Cell suspension was washed in PBS plus 2% fetal bovine serum and 10 mM HEPES. Then, cells were incubated on ice at the dark with Cy5-conjugated rat Anti-mouse CD31 (Pecam-1) or APC-conjugated anti C-Kit (clone ACK2). After washing, the cells were analyzed in a MoFlo cell sorter.

For RT-PCR, total RNA was isolated from the Wt1^cre-YFP^ cell fraction of the intestines obtained from three E15.5 embryos. The cells were dissociated as described above and purified by cell sorting. RNA isolation was performed using NucleoSpin RNA XS kit (Macherey-Nagel KG, Germany). Two-step RT-PCR was performed with the total RNA obtained with the First Strand Amplification Kit (Roche Diagnostica, Spain). C-Kit PCR amplification was performed with GoTaq Flexi Polymerase (Promega, Madison, USA) and with QuantiTech Primer Assays (Qiagen, Hilden, Germany). RNA from an E15.5 whole embryo was used as positive control, and water instead of cDNA was the negative control.

## Results

### Cells of the Wt1 Lineage are Already Abundant Since the Earliest Stages of Intestinal Development

**Figure 1:**
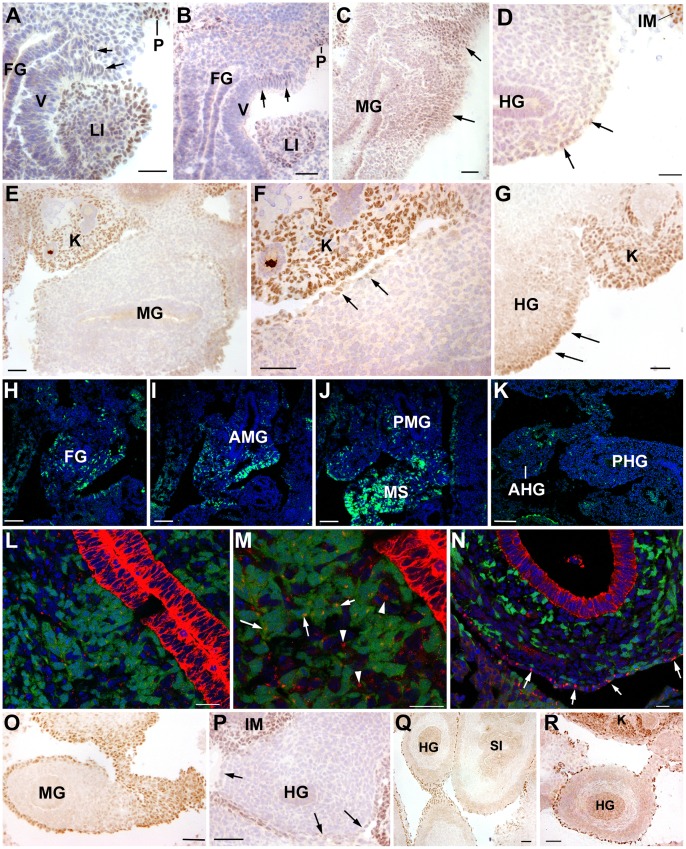
Expression of Wt1 and cytokeratins in early and middle intestinal development and early localization of Wt1^cre-YFP^ cells. A–D. Wt1 immunolocalization in consecutive sections from the same E10 embryo. Wt1 is expressed in a few cells of the coelomic epithelium of the foregut (FG) and in some mesenchymal cells (arrows in A and B). As shown in C and D, the expression becomes more abundant in the midgut (MG) and is weak and restricted to the coelomic epithelium in the hindgut (arrows in D). The Wt1-positive liver bud (LI), parietal coelomic epithelium (P) and intermediate mesoderm (IM) are positive controls. E–G. Wt1 immunolocalization in an E10.5 embryo. Wt1 immunoreactivity is present throughout the coelomic epithelium of the midgut (MG). F shows a higher magnification of the dorsal area of the midgut. Wt1 expressing epithelium is shown by the arrows. Expression in the hindgut has increased as compared with the earlier stage shown in D (arrows in G). Developing kidneys (K) are positive controls. H–K. Wt1^cre-YFP^ cells in consecutive sections of the same E10.5 embryo. Wt1^cre-YFP^ cells are abundant in the foregut (FG) and anterior midgut (AMG), becoming scarcer in the posterior midgut (PMG). Abundant Wt1^cre-YFP^ cells are present in the mesentery (MS) at this level. Ony a few cells are present in the anterior hindgut (AHG) while the posterior hindgut (PHG) lacks of Wt1^cre-YFP^ cells. L,M. Wt1^cre-YFP^ cells in the midgut mesenchyme of an E10.5 embryo. YFP positive and negative mesenchymal cells show a dot-like cytokeratin immunostaining by E10.5 (arrows and arrowheads, respectively in M). The endoderm is strongly cytokeratin immunoreactive. N. Cytokeratin immunostaining has disappeared from the mesenchymal cells by the stage E13.5, and it is restricted to the coelomic epithelium of the intestine (arrows). O–P. Wt1 immunolocalization in an E12.5 embryo. The midgut (MG) intestinal loops contained in the physiological umbilical hernia show a strong Wt1 immunoreactivity (H). However, the hindgut mesothelium is Wt1-negative by this stage (arrows in P). Note the strong Wt1 immunoreactivity of the posterior intermediate mesoderm (IM). Q–R. Wt1 immunolocalization in an E14.5 embryo. All the digestive tract mesothelium is Wt1-immunoreactive, including the small intestine (SI) and the hindgut (HG). K: kidney. Scale bars: A–G, O–R = 50 µm; H–K = 100 µm; L–N = 25 µm.

Wt1 protein expression is present in the coelomic epithelium and mesenchymal cells of the intestine by E10 (Fig. 1A–D), weakly in the fore and hindgut and stronger in the midgut (Fig. 1C). High Wt1 expression is detected by this stage in the coelomic epithelium of the developing liver (Fig. 1A). Wt1 expression becomes uniformly observed throughout all the intestinal coelomic epithelium by E10.5 (Fig. 1E–F) when Wt1^cre-YFP^ mesenchymal cells are already abundant, especially in the foregut and anterior midgut (Fig. 1H, I). Wt1^cre-YFP^ mesenchymal cells are scarcer in the posterior midgut, where many Wt1^cre-YFP^ mesenchymal cells can be seen in the mesentery (Fig. 1J). These cells are much scarcer or even absent in the hindgut (Fig. 1K). On the other hand, cytokeratin immunoreactivity exhibited a dot-like pattern in most intestinal mesenchymal cells, either Wt1^cre-YFP^ or YFP-negative (Figure 1L, M). As discussed below, this pattern suggests an epithelial origin of these cells. This cytokeratin staining is transient and it has disappeared from mesenchymal cells by E13.5 (Fig. 1N).

### Wt1^cre-YFP^ Cells Contribute to the Early Steps of Intestinal Vascularization

**Figure 2:**
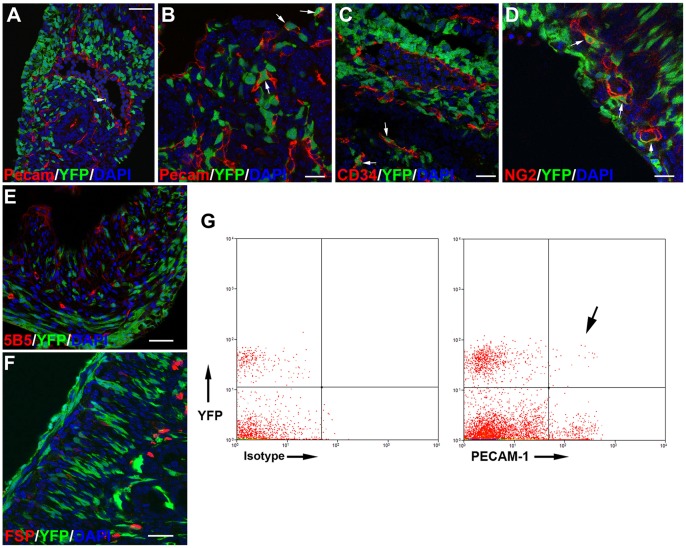
Vascular contribution of the Wt1-expressing cell lineage. A–C. Colocalization of the endothelial markers Pecam-1 (A, B) and CD34 (C) with YFP in intestinal vessels at the stage E11.5. D. Colocalization of the pericyte marker NG2 with YFP at the stage E13.5. E. Immunolocalization of the fibroblastic marker 5B5 at the stage E18.5. This marker does not colocalize with YFP. F. Immunolocalization of the fibroblastic marker FSP1 in a neonate. Colocalization with YFP is not observed. G. Analysis of dissociated intestines from an E11.5 embryo by flow cytometry. In this representative experiment, colocalization of PECAM-1 with YFP (arrow) was found in a 0.3% of the total cells, and in 2.9% of the Wt1^cre-YFP^ cells. Scale bars A, E, F = 50 µm; B,C,D = 25 µm.

**Figure 3:**
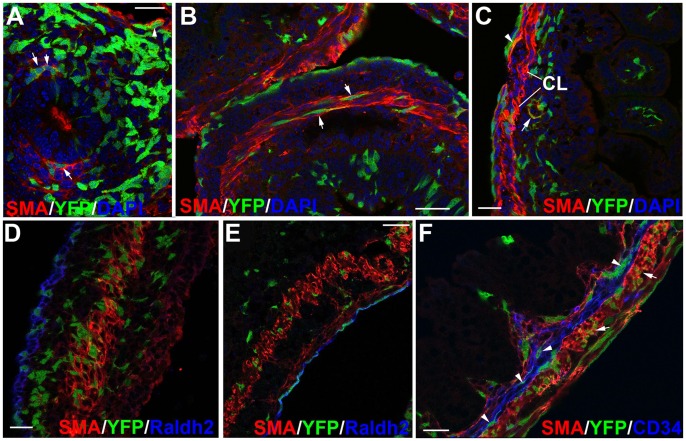
Contribution of the Wt1^cre-YFP^ cells to the visceral smooth muscle and Cajal-like interstitial cells. A. Colocalization of SMCα-actin with YFP in cells ventral to the endoderm at the stage E11.5 (arrows). SMCα-actin+/Wt1^cre-YFP^ cells can be seen in the wall of a large vessel (arrowhead). B,C. Colocalization of SMCα-actin with YFP in visceral circular muscle layer (CL) at E15.5, in transverse and longitudinal section, respectively. A submesothelial cell, positive for both markers, is shown in C by the arrowhead. Note the YFP-positive vessel wall (arrow in C). D, E. The intestinal coelomic epithelium is RALDH2 immunoreactive by E13.5 (D) and E15.5 (E). Note the presence of SMCα-actin immunoreactive cells, probably progenitors of the longitudinal muscle layer, lying behind the RALDH2+ mesothelium in E. F. In this neonate, colocalization of YFP with SMCα-actin is observed in the circular muscular layer (arrows). CD34+ cells are abundant in the submucosal layer, showing thin prolongations. Some of these cells are also Wt1^cre-YFP^ (arrowheads). Note the presence of submucosal SMCα-actin cells forming the innermost muscle layer. CD34 does not colocalize with SMCα-actin. Scale bars = 25 µm.

By E11.5, Wt1^cre-YFP^ cells become very abundant in all the mesodermal layer surrounding the endoderm, including the hindgut (Fig. 2A–C). Most mesenchymal Wt1^cre-YFP^ cells seem to remain undifferentiated throughout these stages. Cells expressing the endothelial markers PECAM and CD34 appear around the endoderm, either isolated or forming small vessels. A few Wt1^cre-YFP^ cells also express these endothelial markers (Fig. 2A–C). SMCα-actin expressing Wt1^cre-YFP^ cells are also found in the vascular walls (Fig. 3A and 3C). Colocalization of YFP with the pericyte marker NG2 is found in embryos at the stage E13.5 (Fig. 2D). However, colocalization of YFP with the fibroblastic markers 5B5 and FSP1was infrequent (Fig. 2E, F).

The colocalization of PECAM-1 with YFP was confirmed by flow cytometry of dissociated intestines at E11.5. This colocalization accounted for 2.9% of all the Wt1^cre-YFP^ cells of the intestine (0.3% of total cells) in the representative experiment shown in Fig. 2G. Colocalization of YFP with endothelial markers was not found in later stages of development.

### Wt1 is Dynamically Expressed in the Intestinal Coelomic Epithelium by Midgestation

Changes in Wt1 immunoreactivity were detected in the coelomic epithelium in embryos of stages E12.5 and E14.5 (Fig. 1O–R). By E12.5, Wt1 expression was high in the mesothelium of the foregut and midgut (Fig. 1O), but the hindgut shows a much reduced Wt1 immunoreactivity (Fig. 1P). By E14.5, however, all the gastrointestinal duct showed a Wt1 immunoreactive mesothelium, including the hindgut (Fig. 1Q, R).

### Wt1^cre-YFP^ Cells Contribute to All the Layers of Visceral Smooth Muscle and also to a Submucosal Layer of Fibroblastoid CD34+ Cells

SMCα-actin expressing cells form a ventral crescent by the stage E11.5 (Fig. 3A) and a continuous ring around the endoderm by E12.5. This ring is located at a middle level, and it is sandwiched between two layers of SMCα-actin negative cells (Fig. 3B). All these layers show a number of Wt1^cre-YFP^ cells, colocalizing with SMCα-actin in the middle layer. In later stages (from E15.5 on) the band of SMCα-actin+ cells becomes more peripherical and the difference between the prospective muscularis layer and the submucosal layer is well established. The longitudinal muscular layer appears by these stages at a submesothelial level, where cells expressing variable levels of SMCα-actin immunoreactivity are appearing (Fig. 3C). This process is more patent in the small intestine, where SMCα-actin+ cells are already arranged in differentiated circular and longitudinal layers by E15.5, while a single, middle layer of SMCα-actin+ cells is still present in the large intestine. The coelomic epithelium shows a strong immunoreactivity for RALDH2 by E15.5, although it seems to be weaker in earlier stages (Fig. 3D, E).

Interestingly, a layer of non-endothelial, CD34+ cells can be observed between the muscular and the submucosal layers (Fig. 3F). CD34+ cells show long filaments and a fibroblastoid appearance. Many Wt1^cre-YFP^ cells are located within this layer and show CD34+ immunoreactivity in their surface.

In addition to the circular and longitudinal muscular layers a loose submucosal SMCα-actin positive cell layer appear in the mucosa of the late embryos and neonates (Fig. 3F). Wt1^cre-YFP^ cells are also integrated in this muscular layer.

### A Number of Wt1^cre-YFP^ Cells Show Expression of the Chloride Channel Anoctamin-1 and Differentiate into c-Kit+ Interstitial Cells of Cajal

**Figure 4:**
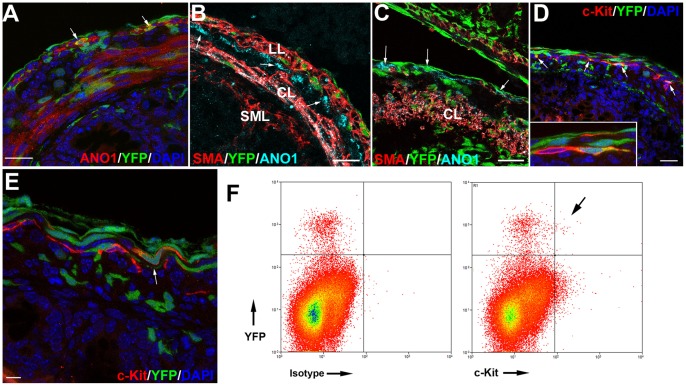
Contribution of Wt1^cre-YFP^ cells to the differentiation of the interstitial cells of Cajal. A. ANO1 immunoreactivity is present within the circular muscle layer and in submesothelial cells from neonates. Colocalization with YFP is observed (arrows). B. Immunolocalization of SMCα-actin and ANO1 in an E18.5 embryo. Colocalization is only detected in the circular muscle layer (CL). However both, the longitudinal (LL) and the submucosal (SML) layers are SMCα-actin positive and ANO1 negative. ANO1 positive, SMCα-actin negative cells (arrows) are present between circular and longitudinal layers, probably representing ICC. C. Immunolocalization of SMCα-actin with ANO1 in a neonate, longitudinal section. Colocalization of ANO1 with YFP is shown (arrows). These cells are negative for SMCα-actin. D,E. Immunolocalization of c-Kit in two neonates. By this stage, c-Kit immunoreactivity labels ICC. Some of these cells are also Wt1^cre-YFP^ (arrows). A c-Kit+/Wt1^cre-YFP^ putative ICC connected to a c-Kit+/YFP- cell is shown in the insert. F. Flow cytometry analysis of cells obtained from the dissociation of an intestine from an E15.5 embryo. Colocalization of c-Kit with YFP (arrow) was found only in 0.03% of all the cells, but the percentage of the Wt1^cre-YFP^ cells that were also c-Kit+ was 3.8% in this representative experiment. Scale bars 25 µm.

Cells from the muscular layer, especially the circular one, show anoctamin-1 (ANO1) immunoreactivity (Fig. 4A–C). This immunostaining becomes strong by E16.5. Colocalization of YFP with ANO1 is observed in many cells of both, the circular and the longitudinal layer.

Colocalization of ANO1 and SMCα-actin is frequent in the circular layer, especially by the E16.5 stage. Colocalization is more scarce in the longitudinal layer and completely absent in the submucosal muscular layer, where all cells are SMCα-actin+/ANO1- (Fig. 4B). In neonates, the colocalization of these markers is very occasional, suggesting a decrease of ANO1 immunoreactivity in muscular cells (Fig. 4C). ANO1+/SMCα-actin- cells are mainly found in the space between the circular and longitudinal layers, the place where the myenteric plexus is developing (Fig. 4B, C).

**Figure 5:**
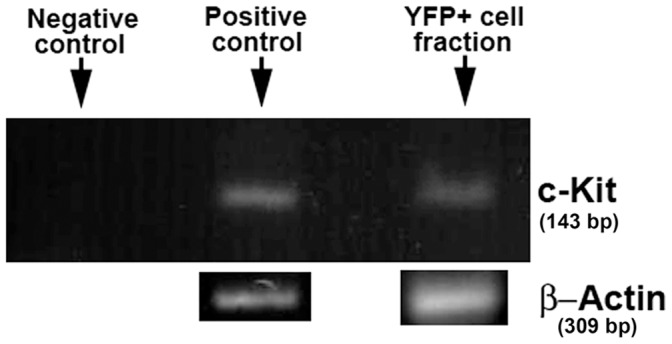
RT-PCR analysis of c-Kit expression in the intestinal Wt1^cre-YFP^ cell fraction. This fraction was purified by cell sorting of the dissociated intestine of three E15.5 embryos. Positive control: cDNA from a whole E15.5 embryo. Negative control: water instead of cDNA.

In neonates, the ICC marker c-Kit was detected in cells from the submesothelial layer and also in cells located between both muscular layers. The elongated morphology of these cells was typical of the ICC. Some of these c-Kit+ cells were also Wt1^cre-YFP^ and appeared closely connected with other c-Kit+/YFP- cells (Fig. 4D, E). Colocalization of c-Kit and YFP was confirmed by flow cytometry of a dissociated intestine from an E15.5 embryo. As shown in the representative experiment shown in Fig. 4F, 3.8% of all the Wt1^cre-YFP^ cells were also c-Kit immunoreactive. Furthermore, expression of c-Kit was detected by RT-PCR in Wt1^cre-YFP^ cells purified by cell sorting of dissociated intestines obtained from E15.5 embryos (Fig. 5).

### Wt1^cre-YFP^ Cells are Transiently Detected within the Endodermal Epithelium and they are Released to the Gut Lumen

**Figure 6:**
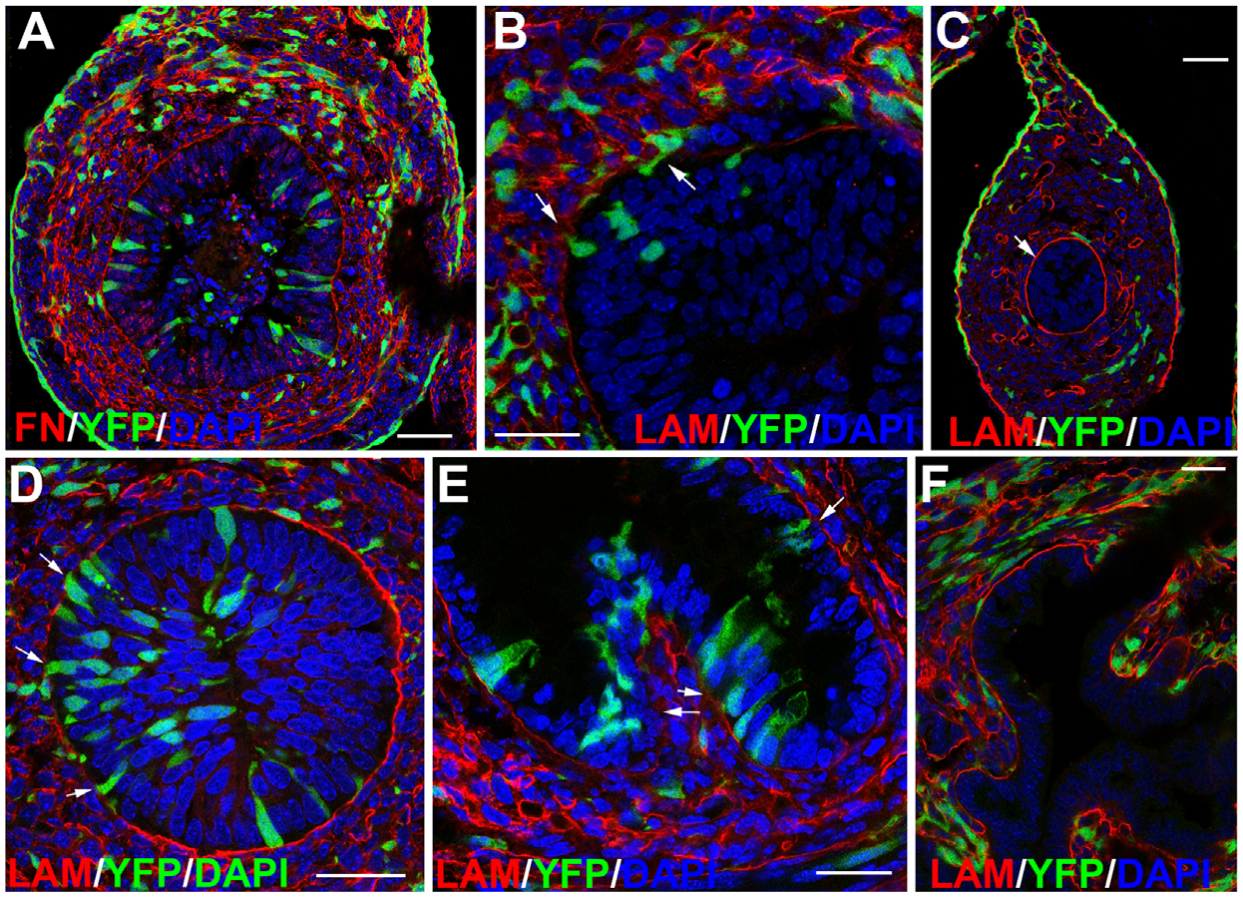
Presence of Wt1^cre-YFP^ cells in the endoderm. A. Fibronectin immunoreactivity is high in the prospective submucosal area. Note the presence of Wt1^cre-YFP^ cells inside the endodermal mucosa, as well as into the intestinal lumen. Many luminal cells show pycnotic nuclei suggesting cell death. B-D. Sections obtained from an E13.5 embryo. Wt1^cre-YFP^ cells seem to be migrating into the endoderm through discontinuities of the basal lamina revealed by lack of laminin immunoreactivity (arrows in B and D). However, at more posterior levels of the same embryo (C) the basal lamina of the endoderm (arrows) is continuous and no Wt1^cre-YFP^ cells are present in the mucosa. E,F. Laminin immunoreactivity shows a continuous endodermal basal lamina in areas lacking of Wt1^cre-YFP^ cells within the mucosa (F, neonate). However, where Wt1^cre-YFP^ cells are still present in the endoderm, the lamina basal seems to be still discontinuous (arrows in E, E18.5 embryo). Scale bars = 25 µm.

By midgestation Wt1^cre-YFP^ cells are present in the gut endoderm. This event starts by E12.5 in the dorsal and anterior part of the gut, and spreads along all the digestive tube (Fig. 3B, 6A, B, D). Some Wt1^cre-YFP^ cells are apparently migrating through the basal lamina of the endoderm. Migration of Wt1^cre-YFP^ cells can be boosted by the high expression of fibronectin in the submucosa (Fig. 6A). In the areas where this phenomenon is observed, the basal lamina of the endoderm shows discontinuities, as suggested by the decrease of laminin immunoreactivity (Fig. 6B, D). However, areas of the mucosa where Wt1^cre-YFP^ cells were absent from the epithelium show an intact, laminin-immunoreactive basal lamina (Fig. 6C). Wt1 immunostaining did not revealed Wt1 expression in the endoderm throughout these stages (Fig. 1J–L). Furthermore, the study of homozygous ROSA26R-EYFP embryos showed no YFP-expressing cells in the digestive tract or elsewhere, thus discarding an artifact due to spontaneous recombination in absence of cre-recombinase (not shown).

Wt1^cre-YFP^ cells apparently delaminate from the gut endodermal epithelium and they are released to the intestinal lumen from E15.5 on, as suggested by some images. In the lumen, Wt1^cre-YFP^ cells show pyknotic and fragmented nuclei, indicating cell death (Fig. 6A). Wt1^cre-YFP^ cells become progressively more scarce in the endodermal mucosa by late developmental stages, and they have disappeared from the intestinal epithelium of neonates. The basal surface of the mucosa is still laminin-negative in some areas, especially where Wt1^cre-YFP^ cells are still present (Fig. 6E). However, in neonates, laminin immunoreactivity is continuous along all the basal lamina of the mucosa (Fig. 6F).

### Wt1^cre-YFP^ Cells Express FGF9 and PDGFRα

**Figure 7:**
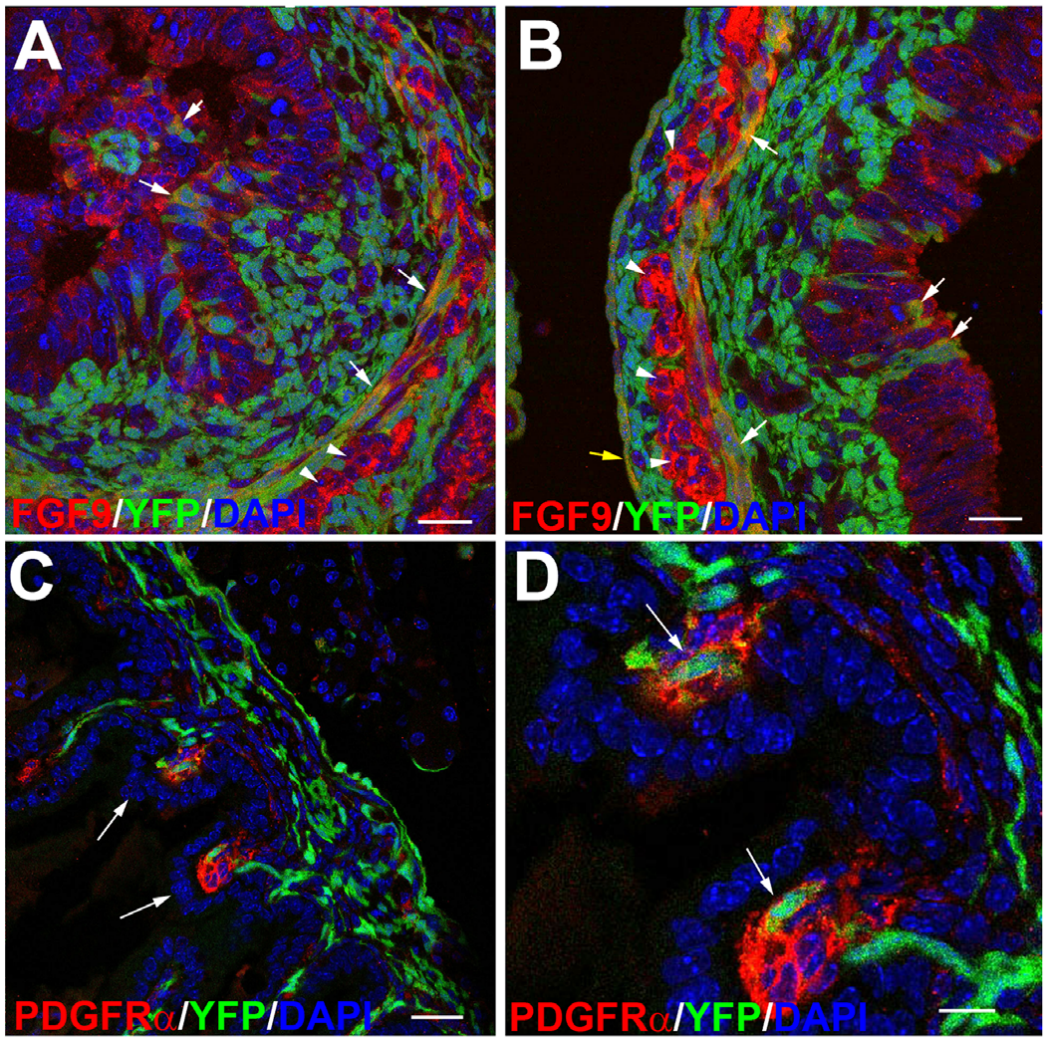
Localization of FGF9 and PDGFRα in the developing intestine. A,B. FGF9 immunoreactivity in the intestine of E15.5 embryos. Colocalization with YFP is observed in cells of the circular muscle layer as well as in Wt1^cre-YFP^ cells within the endodermal mucosa (white arrows). Note the large FGF9+ cells, probably neural, between the circular and the longitudinal muscle layers (arrowheads). The coelomic epithelium is also FGF9+ (yellow arrow in B. C,D. Immunolocalization of PDGFRα in the small intestine of an E18.5 embryo. Positive mesenchymal cells are located within the developing villi (arrows in C). Colocalization of PDGFRα with YFP is shown at higher magnification in D (arrows). Scale bars A–C = 25 µm; D = 10 µm.

FGF9 immunoreactivity is strong in large cells located between the circular and longitudinal muscle layers (Fig. 7A, B) of late embryos. These cells are probably neural, given their strong β-tubulin immunoreactivity (not shown). Immunoreactive cells can also be observed in the coelomic epithelium and in both muscle layers. YFP/FGF9 colocalization is observed in the coelomic epithelium, circular muscle layer and also in the Wt1^cre-YFP^ cells located within the endodermal epithelium (Fig. 7A, B).

The tyrosine-kinase receptor PDGFRα was also expressed by mesenchymal cells (including Wt1^cre-YFP^ cells) within developing villi of the small intestine (Fig. 7C, D) with a strongest immunoreactivity just in the tip of the villus.

## Discussion

In recent years, the study of coelomic epithelium-derived cells lining developing organs has revealed important roles in organogenesis, as described in the introduction. The case of the intestine seems not to be different. The existence of an EMT of the coelomic epithelium lining the intestine, and the importance of the Wilms’ tumor suppressor gene product as a lineage marker of the coelomic-derived cells had already been demonstrated. Our findings have revealed a substantial contribution of the Wt1-expressing cell lineage to the development of multiple intestinal tissues including some, such as the Cajal and Cajal-like interstitial cells, whose developmental origin was poorly known.

Wt1 expression in the coelomic epithelium of the gut starts by E10. It is conceivable that this early activation of Wt1 in the splanchnopleural coelomic epithelium triggers the expression of Snail, a main effector of the EMT [4] promoting the transformation of epithelial cells into mesenchyme. In fact, the embryonic gut already contains an abundant population of Wt1^cre-YFP^, coelomic-derived mesenchymal cells at least since E10.5, first in the fore and midgut, and later in the hindgut. The epithelial origin of these cells is confirmed by the transient, dot-like cytokeratin immunoreactivity. The presence of collapsed cytokeratin filaments in mesenchymal cells is an evidence of their recent epithelial origin [16]. Interestingly, the expression of the Wt1 seems to be highly dynamic, as suggested by the downregulation observed in the hindgut by E12.5 (Fig. 1P). At later stages of the development this expression is recovered, and thus the whole coelomic epithelium of the gastrointestinal duct is highly Wt1 immunoreactive by E14.5. This upregulation of Wt1 expression seems to be unrelated with a new epithelial-mesenchymal transition, and it precedes to an upregulation of Raldh2, the main mesodermal retinoic acid (RA) synthesizing enzyme, and the differentiation of the longitudinal muscle layer. Raldh2 is a target of Wt1 [17] and RA promotes smooth muscle differentiation in mesenchymal stem cells [18]. Thus, the late expression of Wt1 could be related with the differentiation of the longitudinal musculature through a RA-mediated signaling mechanism.

Only during the earliest stages of the intestinal development Wt1^cre-YFP^ cells were able to differentiate into endothelial cells. The sharp decrease of the proportion of endothelium originating from the Wt1-expressing cell lineage can be explained by the recruitment of angioblasts or circulating endothelial progenitor cells. Anyway, our observation supports the concept that cells derived from the coelomic epithelium have vasculogenic potential. This observation correlates our findings with the roles played by cells derived from the epicardium during cardiac development [4]. In both cases, coelomic cells express Wt1, undergo an EMT and give rise to a multipotential mesenchyme, contributing to organ vascularization. We think that this can be a generalized mechanism for vascularization of developing viscera, based on the vasculogenic potential of the mesenchymal cells derived from the coelomic epithelium, a potential that seem to be locally activated as the visceral primordia develop. Besides the vasculogenic fate, the multipotentiality acquired by the cells derived from the coelomic epithelium would allow these cells to differentiate in organ-specific derivatives, such as Cajal interstitial cells, as shown in this paper, or Sertoli cells in the testicle [7].

Smooth muscle differentiation starts in the ventral aspect of the digestive tract. A complete muscular ring is observed by E12–13. Later, an outer, submesothelial layer of SMCα-actin positive cells appears. Our observations point to an independent origin of both muscle layers. Circular arrangement of the muscle cells is already evident when the submesothelial SMCα-actin positive cells have not yet appeared. On the other hand, SMCα-actin immunoreactivity is weak and scattered in submesothelial cells before the appearance of the submesothelial layer. Furthermore, expression of markers seems to be different among both layers. For example, Anoctamin-1 (ANO1) is strongly expressed by E16.5 in the circular layer, but not in submesothelial mesenchyme. Thus, the middle SMCα-actin positive ring of cells seems to contribute only to the circular muscle, being the longitudinal muscle layer a result of the differentiation of submesothelial mesenchymal cells, possibly related with the expression of RALDH2 in the coelomic epithelium, as discussed above. An early differentiation of the circular layer in mice had been also suggested [19], [20], although the differentiation of circular and longitudinal cell layers is described as simultaneous in humans [21]. Finally, the origin of the third muscular layer, called *muscularis mucosae*, seems to be also independent of the other muscular layers. It appears in late developmental stages and neonates and it never expresses ANO1.

Wt1^cre-YFP^ cells contribute to the smooth muscle of all these muscle layers. Wt1^cre-YFP^ cells also express the ANO1 marker, mainly by E16.5, although this colocalization is rare in neonates, where ANO1 immunoreactive cells, most probably progenitors of ICC, have become scarcer and they localize preferentially between the circular and longitudinal muscle layers. This pattern of differentiation is consistent with the descriptions made on human foetuses [22].

We think that our results strongly support the hypothesis of an origin of the ICC from a common progenitor shared with the lineage of the visceral smooth muscle [15]. ANO1 is a calcium-dependent chloride channel and its expression has been associated with the generation of ICC slow waves. ANO1 labels all classes of ICC and represents a highly specific marker for ICC in mouse and human tissues [23], [24]. Probably, the ANO1+/SMCα-actin+ cells of the circular layer can lose one of these markers differentiating either in ICC or visceral smooth muscle. It is important to remark that c-Kit, another ICC marker, is expressed in the circular layer by midgestation [19] supporting this possibility. The ANO1+/SMCα-actin negative cells observed in the developing longitudinal layer are an alternative source. Anyhow, since some ANO1+/SMCα-actin negative cells are also Wt1^cre-YFP^, and also given the YFP/c-Kit immunocolocalization observed in presumptive ICC and the expression of c-Kit detected by RT-PCR in purified Wt1^cre-YFP^ cells, we can conclude that cells from the Wt1 lineage are involved at least in the earliest stages of ICC differentiation.

We have also observed a cell population, containing an important component of Wt1^cre-YFP^ cells, that is constituted of cells with long prolongations and characterized by CD34 immunoreactivity, lacking of SMCα-actin, ANO1 and c-Kit expression. These cells are preferentially located in the submucosal layer. This population probably correspond to the CD34+ Cajal-like or fibroblast-like cells described by other authors [25], [26]. This cell population decreases in some motility disorders such as chronic intestinal pseudoobstruction, suggesting some role related with ICC function [27]. The relation of these CD34+ cells with the so-called intestinal “telocytes” [28] is unclear, since telocytes have been mainly described on morphological criteria in multiple tissues, but their immunophenotyping is needed of clarification.

Colocalization of YFP with PDGFRα into the developing villi demonstrates that cells from the Wt1-expressing lineage are also involved in villus morphogenesis. In fact the clusters of PDGFRα positive cells had been already described, and the essential role played by PDGF-A and its receptor PDGFRα was well known [29]. Together with the expression of FGF9 (discussed below) this observation suggests that the cells derived from the coelomic epithelium are not only contributing to a number of tissues but also playing a signaling role for intestinal tissue morphogenesis.

It is interesting to remark that colocalization of Wt1^cre-YFP^ cells with the fibroblast markers 5B5 (prolin hydroxylase subunit) and FSP1 was rare in the embryos studied. This might reveal a lineage restriction of the Wt1^cre-YFP^ cells towards the muscular/vascular rather than to the connective tissue, but it is also possible that the timing of differentiation towards fully-differentiated, collagen-producing cells be delayed in Wt1^cre-YFP^ cells in relation to other developmental fates. Thus, the ontogenetically older splanchnopleural cells could be predominant in the differentiation of the early fibroblasts. Studies in young Wt1^cre-YFP^ mice would be necessary to confirm this.

Finally, our most intriguing observation has been the presence of Wt1^cre-YFP^ cells within the endoderm, given the lack of expression of Wt1 in this tissue. The signs of migration of Wt1^cre-YFP^ cells throughout discontinuities of the basal lamina suggest that mesodermal cells can move throughout the endoderm coinciding with the transition from the pluristratified to the columnar epithelium. The process starts by E12.5 and seems to decline after E16.5. What seems to be clear is the release of all the mucosal Wt1^cre-YFP^ cells to the intestinal lumen where they show signs of cell death. We have found only a paper describing the migration of mesenchymal cells through the gut endoderm in the human fetus, by stages corresponding to our observations in mouse [30]. The question remains open but we think that it deserves further research. The hypothetical migration seems to involve degradation of the endodermal basal lamina. This could be physiologically relevant since proliferation and differentiation of the intestinal epithelium is dependent on paracrine signals from mesenchymal cells. If migration of mesenchymal cells into the endodermal compartment were confirmed, this would allow a paracrine release of these factors within the endoderm. FGF9 has been related with intestinal differentiation [31], [32], and we have observed intraendodermal Wt1^cre-YFP^/FGF9+ cells. Significantly, cardiac ventricle proliferation and differentiation is promoted by FGF9 secreted by epicardially-derived cells (the developmental equivalent of the coelomic-derived cells of the intestine) migrating throughout the myocardiocytes [2].

In summary, cells from the Wt1 lineage seem to play multiple and dynamic roles in intestinal development. Furthermore, the cells from the Wt1 lineage seem to show no difference in their distribution or developmental fate with respect to other splanchnopleural mesodermal cells. In contrast with previously published data [9] we show evidence that cells derived from the coelomic epithelium can differentiate into endothelium, vascular and visceral smooth muscle, Cajal and Cajal-like interstitial cells. Thus, it would suggest that the only difference between the Wt1-expressing lineage cells and other splanchnopleural cells is their late origin. It is conceivable that the role of Wt1 would be to promote and to temporally extend the EMT of the splanchnopleura in order to increase the population of mesodermal cells that contribute to different intestinal tissues. The differentiation of this pool of splanchnopleural cells would be dependent from local signals. These findings will raise many new questions whose investigation can contribute to our knowledge of the development of the digestive tract and its congenital motility disorders.
